# Usability Study of a Wireless Monitoring System among Alzheimer's Disease Elderly Population

**DOI:** 10.1155/2014/617495

**Published:** 2014-05-20

**Authors:** Stefano Abbate, Marco Avvenuti, Janet Light

**Affiliations:** ^1^Institute of Informatics and Telematics, National Research Council, Via G. Moruzzi 1, 56124 Pisa, Italy; ^2^Department of Information Engineering, University of Pisa, L. Lazzarino 1, 56122 Pisa, Italy; ^3^Department of Computer Science & Applied Statistics, University of New Brunswick, Saint John, NB, Canada E2L 4L5

## Abstract

Healthcare technologies are slowly entering into our daily lives, replacing old devices and techniques with newer intelligent ones. Although they are meant to help people, the reaction and willingness to use such new devices by the people can be unexpected, especially among the elderly. We conducted a usability study of a fall monitoring system in a long-term nursing home. The subjects were the elderly with advanced Alzheimer's disease. The study presented here highlights some of the challenges faced in the use of wearable devices and the lessons learned. The results gave us useful insights, leading to ergonomics and aesthetics modifications to our wearable systems that significantly improved their usability and acceptance. New evaluating metrics were designed for the performance evaluation of usability and acceptability.

## 1. Introduction

Healthcare technology using wireless sensors has reached a high level of maturity and reliability and hence these devices are now being deployed in homes/nursing homes for use in managing people's health. To take full advantage of the penetration of these pervasive systems in people's well-being and reap their full benefits, the technologies must be minimally invasive and must be accepted by users willingly.

A necessary condition for acceptance is the awareness of benefits to the user population in using the system. Since young adults are well acquainted with modern sensor devices, they willingly accept the introduction of new technologies in their care process. In contrast, among the elderly, who are the main beneficiary population of these monitoring devices, there is still reluctance on their part to use them. Even though an increasing number of the elderly are aware of the advantages of a pervasive health monitoring system, they rarely understand how it works.

With the increase in Alzheimer's disease (AD) among the elderly population, there is a crucial need for technological support in their care process. Since there is no cure yet to reverse the cognitive decline among these individuals, technology could contribute to safely perform their normal living activities.

Unfortunately, people affected by AD have difficulties in understanding their health conditions, and the use of a device or systems that could help in their day-to-day activities. Some of them have a different perception of objects and are prone to forget using them or be adamant in not using them. In our initial study we found that even the simplest interactive devices such as wristband buttons or call buttons to alert a caregiver in an emergency situation are unlikely to be used by them.

Despite the technological maturity of healthcare devices and networking, little effort has been done to assess their usability and acceptability before deployments in homes and nursing homes. Health monitoring platforms developed so far have mainly focused on the functionalities using specific sets of sensors, vendor specific software, and protocols, for which usability issues have not been sufficiently addressed.

We addressed this gap by undertaking this exploratory study on how to increase the usability and acceptability of our wearable monitoring system to a small group of elderly affected by AD. In the study, we used the wireless accelerometer and electroencephalograph (EEG) logger integrated in our minimally invasive monitoring sensor (MIMS) system [1], with the aim of detecting possible falls and their preconditions. The wireless monitoring system used did not require any interaction with the subjects. However, the living environment should include a network infrastructure with wired alert systems to connect to central nurse care stations and/or wireless networks to hand-held devices carried by the nurses.

We defined ad hoc usability and acceptability parameters and evaluated them during a month long field test with long-term nursing home residents. The results gave us some important insights, leading to ergonomics and aesthetics modifications to our system that significantly improved its usability and acceptance.

## 2. Related Work

A number of systems engaged in health monitoring are surveyed here to compare our approach to those of others. Though every system studied here has been in deployment for a long period of time, it was disappointing to observe that none of them reported about user's acceptability of the system. Only technology descriptions were made public about these systems.

For example, Cao et al. survey of enabling technologies for wireless body area networks [2] discussed the network characteristics, such as the type of wireless connection (Bluetooth, ultrawideband, ZigBee) and path loss of the signal sent by body sensors according to their placement on the body and to the radio frequency used. Performance evaluation and their usability study results are missing.

A wearable monitoring system called SATIRE [3] collects the motion and location information of a subject. SATIRE requires sensors inserted in the garment worn by the user without the need of user's interaction. The sensors collect and store data locally. Periodically, the data is uploaded to a base station for further analysis and archive. The paper presents the design which includes a layered architecture (for both the base station and the sensors). Real-world testing and adaptability study of this system are not known.

The MIThril LiveNet system [4] is another distributed mobile system for real-time monitoring and analysis of the health status of an individual. The MIThril architecture offers many features to perform distributed sensing, classification in real-time, and context aware applications. It makes use of a PDA which should be worn by the patient at all times: body worn sensors send data through a network infrastructure for exchange of information and a machine learning infrastructure is used for classification of gathered data. Again, the usability and adaptability results are not reported.

Finally, the experience gained by the authors in developing fall monitoring systems presented in [5, 6] definitely proved that a usability study is important to provide the required metrics to evaluate the performance of a health monitoring system in real-world applications, especially with the elderly population.

## 3. Materials and Methods

**Figure 1:**
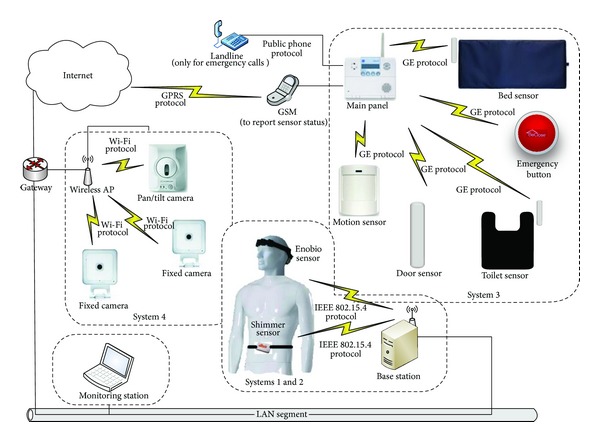
System overview.

The equipment we used for the usability study was based on the MIMS system described in [1]. We developed MIMS with the aim of providing a flexible and scalable platform for building a comprehensive and customizable health monitoring system, which guarantees interoperability among different sensor systems. MIMS can be easily integrated with any wireless communication system already in place and with any existing networked alert system.  Figure 1 shows the complete monitoring system, consisting of four sensing systems.

**Figure 2:**
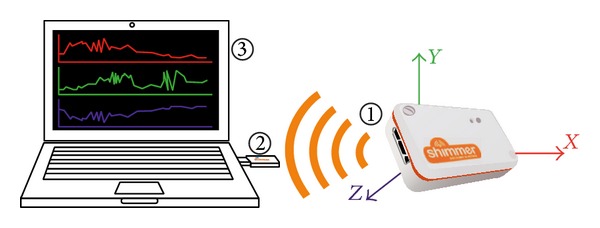
System 1—fall detector. (1) Shimmer 2R with accelerometer. (2) 802.15.4 receiver. (3) Base station.

System 1 is a fall detector. It consists of a wireless sensor node (based, in our case, on the Shimmer 2R platform [7]) able to sense human movements using an embedded accelerometer. The microcontroller (MSP430 family) can perform on-chip analysis and communicate with a base station using a Bluetooth module or IEEE 802.15.4 radio, as shown in Figure 2. Being battery powered, small, and lightweight, the device can be conveniently worn near the waist. The device runs a simple yet very reliable algorithm for fall detection described in [6]. In a nutshell, every time the acceleration reaches a given threshold, samples belonging to a fixed time window around the event are sent to the base station, which in turn analyzes the pattern of accelerations and decides whether the event was due to an activity of daily living (i.e., a false alarm) or to a real fall. In the case of a real fall, the system informs the caregiver through the alert system.

**Figure 3:**
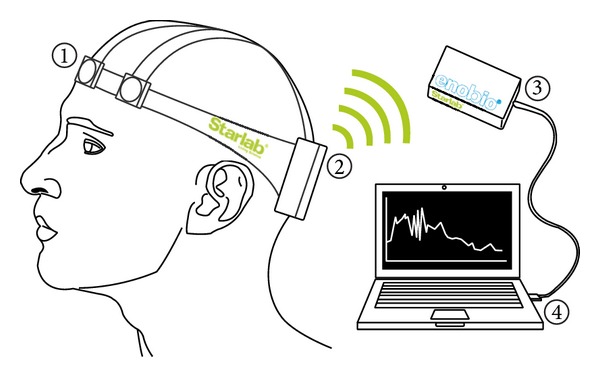
System 2—EEG analyzer. (1) Electrodes. (2) Battery pack/transmitter. (3) 802.15.4 receiver. (4) Base station.

System 2 consists of a wireless electrophysiology sensor (based, in our case, on the Enobio platform [8]) which is able to capture the brain activity of a person in real time. Four digital electrodes are attached to the Enobio communication module and placed in the Enobio headband. Data from such electrodes/channels are wirelessly transferred to a base station using the IEEE 802.15.4 low power radio standard. The base station is represented by the Enobio USB receiver connected to a PC. Two wired ear clip electrodes (potential ground and potential ground feedback) act as references for sensed signals. Enobio (see Figure 3) is worn like a hat and can record not only brain activity but also heart activity through an electrocardiogram (ECG) and eye movements through an electrooculogram (EOG). The Enobio software is a Java application that allows (i) wireless communication between the Enobio and PC; (ii) data recording; (iii) data display; (iv) forwarding of data to other clients. Data is coded as simple ASCII file of tab delimited columns and can also be exported to the very common scientific format EDF (European Data Format). We used System 2 to analyze EEG potentials during the different stages of sleep, with the aim of studying brain signal patterns preceding a fall.

System 3 is composed of ambient sensors such as pressure pads, which are placed in the care environment to monitor lying on a bed or sitting on a chair, and volumetric motion detectors, which cover an area of 10.7 × 9.1 meters and are used to detect motion and activities such as entering/leaving a room. Door sensors are placed on the top of doors, windows, and drawers to detect when they are opened or closed; the toilet mat sensor detects both presence on or near the toilet to monitor bathroom activity and potential safety issues such as falls near to the toilet; emergency buttons can be placed where accidents are likely to happen; they are 7.6 centimeters in diameter; when pressed, they send an alert to the caregiver or to an emergency response service. The main panel acts as a base station and collects data from sensors exploiting the radio channel and a proprietary protocol (General Electric); the base station has battery backup and is equipped by a GSM transceiver and an interface to the landline.

System 4 is a camera-based monitoring system using the internet to continuously stream the video recording human activity (visual motion detection) over a selective region and over a specified observation period (e.g., at nighttime). Two types of cameras have been used: a fixed wireless camera (ADCV510) and a pan/tilt camera (ADC-V610PT). They both have the live resolution options 640 × 480, 320 × 240, and 176 × 144, whereas the recording resolution options are 640 × 480 and 320 × 240; the recording compression is based on MPEG-4. The video motion detection can be configured with three different windows having adjustable sensitivity and thresholds. The streaming of video to the internet relies on a wireless Wi-Fi router and standard encryption (WEP, WPA, or WPA2); cameras can also be connected using a standard Ethernet connection. They are designed to work with the* Alarm.com* hosted video service which provides a surveillance solution. High-quality live and recorded videos are available to customers through web browsers or via mobile apps. Users can set and recall “preset” views or manually pan and tilt the camera remotely.

In the case of resource constraints for sensing, processing, storing, and communicating, some computational operations are delegated to the Virtual Hub, which is a base station running on a smart phone environment. The Virtual Hub receives data from the subsystems and is connected to the local healthcare information system through a friendly graphical user interface. A thorough description of the MIMS platform can be found in [1].

System 1 and System 2 are examples of active monitoring sensor system (AMSS). As they are based on wearable devices, they offer advantages in terms of continuous monitoring, cost, and efficiency. However, their acceptability strongly depends on the level of usability. System 3 and System 4, instead, were not considered in this study as they are environmental systems, whose major concerns are intrusion and privacy rather than usability.

### 3.1. Measuring Usability and Acceptability

According to the human engineering principles [9], the design of a system must follow the users' needs, fear, mental models, self-learning ability, social behavior, lifestyle, and fashion tastes. In fact, an accurate knowledge of end users can be achieved only by observing them closely.

In the case of monitoring people with AD/dementia living in a home or a nursing home, providing suitable care to them requires 24 × 7 continuous monitoring of their everyday activities [10]. Some of their regular daily activities are walking in a corridor, watching television, and, with the help of caregivers, having breakfast, lunch, dinner, and medication; some subjects have a small nap in the afternoon. They are prone to disorientation and wandering at any time of the day or night, and statistics show that they are more prone to falls compared to general elderly population [11]. To compensate psychomotor deficiencies, variant medical equipment is used such as canes, crutches, and wheelchairs. However, not all the subjects are able to understand (or remember) that they need to use them during their walking activity to prevent fall.

In this context, we define usability as the level at which a device can assist a user without interfering with his/her normal activities of daily living. Acceptability is defined as the constraints which guide the designer to realize factors that satisfy one's need and therefore people's willingness to use. The following are the evaluation criteria we developed to measure usability and acceptability:

1. willingness to use (WTU),
2. easiness to learn (ETL),
3. time to accept (TTA),
4. willingness to keep (WTK),
5. number of errors (NOE) due to incorrect interactions,
6. level of satisfaction (LOS),
7. interference with activities of daily living (IWA).

The ranking of evaluation criteria is shown in Table 1.

Results of a Nielsen's research [12] have shown that a usability study can be suitably performed with up to 5 subjects, because the behavior of users does not change significantly as their number increases. This matches the obvious fact that performing small tests does not require huge investments for devices, and it makes the test feasible when, as in our case, it is done on a very critical population such as the elderly affected by AD. Of course, this does not hold for statistical studies, but here we are interested in only qualitative results, as the goal is to gain insights for improving the design of wearable devices based on feedback received by users to increase the usability and adaptability.

Based on these observations, we tried to get the maximum benefit-cost ratio by delivering the usability and acceptability test to four subjects affected by AD with advanced age. The four subjects involved in the test were from 75 to 92 years old. All of them were at staggering stages of dementia progression and associated abnormal behaviors, thus limiting the usability study. In particular, AD subjects were at levels 5/6 of Reisberg stage and they resulted below a score of 12 out of 30 (severe cognitive impairment) of the MMSE (Folstein test). All the patients were in long-term care.

It should be mentioned here that, for 24 × 7 monitoring of AD individuals, a number of trials had to be conducted before a full data collection is accomplished, with the result that some of these tests could not be completed in consecutive days. Some contributing factors to the difficulty in monitoring were age, disease, and associated behavior. Nevertheless, the observations were invaluable.

## 4. Results and Discussion

Table 2 summarizes the results of the study using Systems 1 and 2 of the MIMS platform. Experiences and reactions of the subjects in adapting to the wearable devices and to the monitoring sessions are reported in terms of the usability and acceptability criteria described above, together with observed reactions from the subjects.

In the overall, the study showed that with a few modifications to the way devices are placed, colored, or integrated with clothing, and after some convincing story about the importance of wearing the devices, AD individuals eventually wore and benefited from the monitoring technologies. Since the Shimmer sensor has to be placed on the waist, System 1 achieved higher usability and acceptance than System 2. From the study, we found that integrating the sensor with clothing, so that it could be considered as an everyday accessory, made it better accepted. In doing so, the device must be prevented from choking the individual, and the difference in dress code between women and men must be considered. Since the device is sensitive to movements, careful wearing practices must be observed while placing/removing the device, and touching, meddling, or breaking the device should be prevented during specific activities such as lying on a bed (during the afternoon nap) or sitting on a chair.

Therefore, we adopted the following two solutions.

**Figure 4:**
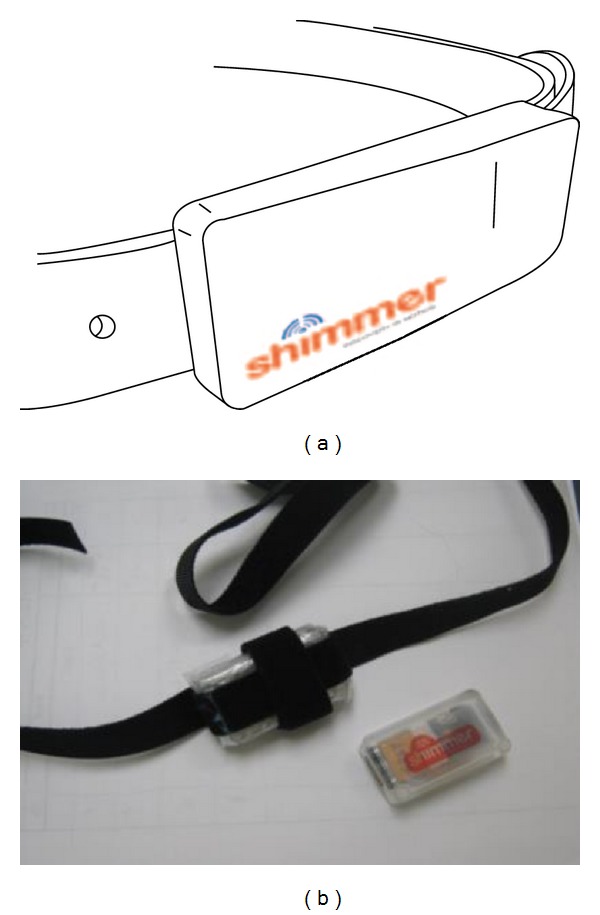
Shimmer integrated into a belt buckle (a). Shimmer attached to a Velcro stripe (b).

1. The device was integrated onto a belt buckle (see Figure 4(a)). A leather style gave the buckle a retro aesthetic that made it suitable for both men and women.
2. The device was attached to a Velcro stripe belt. Two small stripes were crossed to hold the device firm and properly placed on the waist (Figure 4(b)). For women, the device was hidden under the shirt/vest.

A significant effort was necessary to improve the usability of System 2, as the Enobio sensor must be worn like a hat during night sleep. The main problem arose from the presence of a bulky battery pack and transmitter on the user's nape, and this initially made it almost impossible to carry out sleep tests. During the study, we repeatedly modified the device in order to improve its acceptability (measured by WTU) and to make it as least intrusive as possible in order to avoid the user feeling embarrassed while wearing it (measured by WTK).

**Figure 5:**
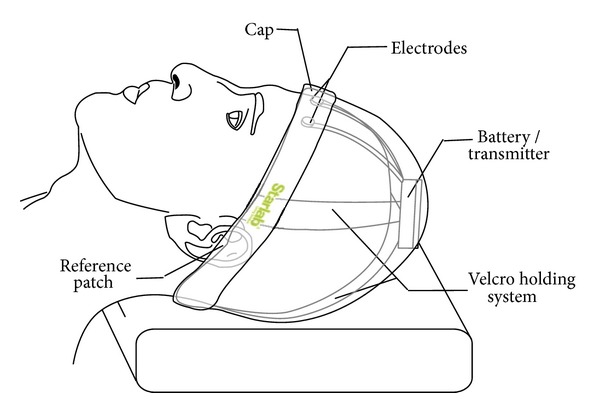
The modified cap was more comfortable during sleeping/going to bed.

To improve the ergonomics of the Enobio sensor, we moved the battery/transmitter from the back of head/neck to the top of the head on a belt. For acceptability, we observed that the elderly enjoy wearing caps in the night to keep them warm; so we worked on the aesthetic side by embedding the sensor in a bonnet style cap with a light texture to prevent sweating (see Figure 5). However, it happened that some users took the hat off before getting into a deep sleep. This problem was partially solved by asking them to wear the hat without the sensing device during daytime. In this way, they got acquainted with wearing the hat and no longer noticed the sensing device was embedded in the hat during the sleep time.

An important factor to be considered here is the color of the device's enclosure. Colors have different impacts and meanings in one's space or environment. Bright colors or color combinations can help visually impaired people in understanding the surroundings. Warm colors such as orange red, pink, yellow, brown, and their shades are favorable for identifying objects. Cool colors such as blue, green, purple, and their shades are useful to give an impression of coolness, discretion, and serenity. The study gave us evidence that when a device comes in one's favorite colors, it is easier to make it acceptable, as it happened with subject 1 to whom we provided the Enobio with a pink cap.

## 5. Conclusions

Technologies applied to healthcare are meant to improve the wellness among people. However, not everyone easily accepts such technologies as designed by engineers. The usability study showed that the design and development of a monitoring device must consider its target users' preferences before it can be broadly deployed. Nontechnical factors depending on both the users and the environment must be considered for quick adaptability and reap the benefit to improved care.

The wireless sensor devices developed within our fall monitoring project were tested to assess their usability among AD elderly in a long-term care home. This rare opportunity gave us many insights leading to positive changes in our system in terms of ergonomics and aesthetics, as well as some modifications to our system architecture. Though the sample size was small due to the complexity in conducting the tests and the difficulty to manage AD subjects for test during day and night, we were able to achieve a qualitative usability assessment.

Patient's unawareness of the system's benefits is a major concern. For example, the Enobio EEG wearable sensor was formerly tested with healthy subjects informed of the sleep study. Even though they reported a slight discomfort, they were always conscious of wearing the hat and often restricted their movements while being in the bed. During the study, our patients showed different reactions. They did not understand about the sleep tests performed. Some of them thought that the hat was to keep them warm. While someone did not move at all in the bed, assuming a supine position for the entire night, others kept removing the hat. As a result, their sleep was interrupted by the testing, as the hat had to be put back to the correct position several times.

The study suggests that ergonomic and aesthetic modifications are necessary to improve the level of usability and acceptability, especially in an elderly user population. Analysis of the users' dress code was fundamental to figure out a comfortable and easily wearable solution. Typically, the elderly are attached to a specific aesthetic dress code, characteristic of their likes/dislikes. They prefer simple, loose, and comfortable dress and therefore the focus should be on a retro style. Unfortunately, such loose dresses make it difficult to put wearable devices close to the body in order to monitor accurately. At the same time, care must be taken such that these devices should not cause itching, rashes, or skin diseases if worn too tight.

From a manufacturing point of view, the devices worn by a patient must be robust and waterproof to avoid accidental damages (e.g., the sensor can fall to a sink full of water, or it can be thrown away or tampered). The device should also provide a switch or a special combination of buttons in order to be activated and deactivated by the nurse before placing it. A battery indicator is another element that would help nurses to identify if the device needs to be charged. The caregivers interface is fundamental to understand how to assign each sensing node to a person, to check the history and general status. In particular, the sensing devices should periodically send a message to signal that they are working correctly and, in this case, they will also provide an update to a localization system.

From the technical point of view, it was found that the deployment of a monitoring system should consider existing communication network infrastructure and the range of sensors in a pretesting phase. Feedback from such testing would enable modifications to system's functions in order to improve its performance and adaptability. For example, in designing the Shimmer-based prototype we initially set the sensors to work within a 100-meter range from the base station, without considering that our subjects were used to walk back and forth along the corridors even far from their bedrooms. After the study, we realized the need of extending the coverage of the sensors for continuous monitoring. A possible solution would have been to adopt a multihop routing protocol, with each node running a message forwarding program besides the monitoring one. However, this would have drastically reduced the nodes' battery lifetime and, in the end, the system's usability. We identified the right solution by distinguishing nodes into two types:* sensing nodes,* worn by the individuals, and* forwarding nodes*, connected to power outlets at fixed locations in the nursing home. Sensing nodes send data to the base station through the forwarding nodes, by selecting the closest node at one-hop distance. Forwarding nodes run a routing algorithm that guarantees no packet loss, which is a critical requirement because the system cannot tolerate undelivered alarms.

As final remarks, even though a cost analysis is beyond the scope of this paper, we would like to stress on the fact that, as argued by analysts addressing the problem of why technology innovation tends to increase the cost of healthcare rather than making things simpler and cheaper [13], the best way technology can save costs is if it is used to better organize the healthcare system. In this sense, systems like the MIMS platform offer an automatic and seamless monitoring system, enabling continuous data collection otherwise very difficult or even impossible to obtain. The bare technological cost of the MIMS equipment used for our experiments, which glues together known and mature technologies, is in the order of 200 USD and 2,000 USD for Systems 1 and 2, respectively. However, we must be aware that the market cost of such systems is driven by healthcare stakeholders other than the research laboratories, whose role is only to propose proof-of-concept, innovative healthcare systems. In commercial use, the entire set of the ambient monitoring systems shown in Figure 1 are being deployed by healthcare companies in homes and nursing homes of some North American provinces, for an approximate cost of 100 USD for a month. So, when deployed in large numbers, there are considerable savings in healthcare cost with manageable equipment cost.

## Figures and Tables

**Table 1:** Usability and acceptability parameters.

Metrics	Levels		
WTU	Low	Medium	High
ETL	Low	Medium	High
TTA	Short	Average	Long
WTK	Low	Medium	High
NOE	None	Few	Many
LOS	Low	Indifferent	High
IWA	None	Low	High

**Table 2:** Behavior of the subjects towards new monitoring systems.

System 1	Subject 1	Subject 2	Subject 3	Subject 4
Day 1	WTU: low	WTU: n/a	WTU: n/a	WTU: high
	ETL: high	ETL: low	ETL: high	ETL: high
	TTA: n/a	TTA: short	TTA: short	TTA: short
	WTK: low	WTK: high	WTK: medium	WTK: high
	NOE: low	NOE: many	NOE: none	NOE: none
	LOS: low	LOS: high	LOS: indifferent	LOS: indifferent
	IWA: none	IWA: none	IWA: none	IWA: none
	Did not want to wear it. After 30 minutes the subject forgot about it and was happy to remove it at the end of day.	No resistance. Then the patient removed it and played with it. At the end, he did not want to give it back.	No resistance.	No resistance. The patient was already using a pouch around the waist.

Day 2	WTU: medium	WTU: high	LOS: none	WTK: medium
	WTK: medium	NOE: few		
	NOE: low		Totally indifferent.	Indifferent.
	LOS: indifferent	Happy to wear it.		
	Light resistance to wear it initially.			

Day 3	WTU: high	NOE: none		
	TTA: long	LOS: indifferent		
	WTK: high			
	NOE: none	Indifferent.		
	Happy to wear it. The family members convinced the subject to wear it saying it was meant for stomach pain relief.			

System 2	Subject 1	Subject 2	Subject 3	

Night 1	WTU: high	WTU: medium	WTU: low	
	ETL: low	ETL: low	ETL: high	
	TTA: n/a	TTA: n/a	TTA: short	
	WTK: low	WTK: low	WTK: medium	
	NOE: few	NOE: high	NOE: none	
	LOS: low	LOS: low	LOS: low	
	IWA: high	IWA: none	IWA: low	
	No resistance at the beginning (the patient loves hats). The patient removed it after some time.	The patient kept for some minutes then removed it. After three times the patient did not want to wear it anymore.	No resistance but the patient did not like it.	

Night 2	NOE: many	WTU: low	WTU: medium	
	IWA: low	WTK: low	WTK: medium	
		NOE: high	LOS: medium	
	The subject wore it but moved it many times.			
		Removed it many times during night.	No resistance.	

Night 3	ETL: medium	TTA: average		
	TTA: average	WTK: medium		
	WTK: medium	NOE: none		
	NOE: none	LOS: indifferent		
	LOS: indifferent			
	The subject was willing to wear it and did not move at all during night.	The subject was monitored from bedside all night to make sure she wore it.		
